# Type II Myocardial Infarction: Predisposing Factors, Precipitating Elements, and Outcomes

**DOI:** 10.7759/cureus.9254

**Published:** 2020-07-18

**Authors:** Bharat Pillai, Sreekrishnan Trikkur, Umar Farooque, Devraj Ramakrishnan, Jyothi J Kakkra, Gayatri Kashyap, Chirag Lalwani, Amirtha B Mani, Jay Vishwanath

**Affiliations:** 1 Neurology, Amrita Institute of Medical Sciences, Kochi, IND; 2 Emergency Medicine, Amrita Institute of Medical Sciences, Kochi, IND; 3 Neurology, Dow University of Health Sciences, Karachi, PAK; 4 Preventive and Social Medicine, Government Medical College, Idukki, IND; 5 Medicine, Amrita Institute of Medical Sciences, Kochi, IND; 6 General Medicine, Amrita Institute of Medical Sciences, Kochi, IND

**Keywords:** coronary insufficiency, type ii myocardial infarction, myocardial infarction, precipitating factors, outcomes, sepsis, heart failure, anemia, electrolyte imbalance, arrhythmia

## Abstract

Introduction

Myocardial infarction (MI) is a subset of the spectrum of the disease known as acute coronary syndrome (ACS), which comprises three distinct entities including unstable angina (UA) and MI with or without ST-segment elevation. However, many clinicians are unaware that MI itself is classified into five types, the most common being type I, followed by type II. Type II MI occurs due to coronary insufficiency not related to acute plaque change in the coronary vasculature. Data available on type II MI is still limited, particularly in the South Asian setting, despite documented poorer outcomes for the same compared to other types. Therefore, we conducted this study as an attempt to outline the predisposing factors, precipitating elements, and possible outcomes of type II MI.

Materials and methods

This prospective study was conducted at a tertiary care hospital in Kochi, Kerala for 12 months. A total of 59 patients of ages 10-99 years, with a final diagnosis of MI based on the levels of cardiac biomarkers and electrocardiography (ECG), no previous history of coronary angiography, thrombolysis, percutaneous coronary intervention (PCI), and non-ischemic conditions producing elevations in cardiac biomarkers were included in this study. Demographic features, cardiac biomarker levels, comorbidities, precipitating factors, foci of sepsis, and outcomes of type II MI were noted. The mean was calculated for age and cardiac biomarkers. The frequency and percentages were calculated for gender, comorbidities, precipitating factors, foci of sepsis, and the outcomes of type II MI.

Results

The mean age of the patients was 69.66 years; 38 (64.4%) patients were males and 21 (35.59%) were females. Mean elevation of creatine kinase myocardial band (CK-MB) was 47.457 IU/L and highly sensitive troponin I (Hs-Trop I) was 8.712 ng/mL. Diabetes mellitus [44 (74.57%)] and hypertension [41 (69.49%)] were the most common underlying patient comorbidities followed by dyslipidemia [38 (64.4%)]. Most of the patients had more than two comorbidities at a time; 33 (55.93%) patients had sepsis, 31 (52.4%) patients had anemia, 29 (49.1%) patients had electrolyte imbalance, 19 (32.2%) patients had respiratory failure, 16 (27.11%) patients had arrhythmia (tachyarrhythmia/bradyarrhythmia), and two (3.3%) patients had postoperative (non-cardiothoracic) stress. Sepsis was originating from the lower respiratory tract in 14 (42.42%) patients, blood in 11 (33.33%) patients, urinary tract in eight (24.24%) patients, and abdomen in six (18.18%) patients. Thirty-four (57.62%) patients had heart failure, 13 (22.03%) had arrhythmias, and 19 (32.20%) patients died.

Conclusions

Type II MI has a high mortality rate, mostly due to heart failure and arrhythmia. Patients with diabetes mellitus and hypertension are at increased risk of type II MI. Sepsis is the most common precipitating factor, primarily originating from the lower respiratory tract, followed by anemia and dyselectrolytemia. Treatment of precipitating factors is the primary way to manage type II MI.

## Introduction

Acute myocardial infarction (MI) is one of the most common critical conditions encountered in the emergency department in any setting. Due to the enormous morbidity and mortality associated with it, along with increasingly sensitive methods of detection and a high index of suspicion clinically, a surge in the number of cases with MI has been reported in the past few years. This has subsequently resulted in an increase in awareness and knowledge about this disease. Despite well-established clinical guidelines and protocols for its management, a significant number of cases still go underdiagnosed and inadequately treated, especially in the Indian subcontinent [1-4].

In spite of the established fourth universal definition of MI, there still persists an uncertainty regarding the diagnosis and treatment of type II as opposed to type I MI. This is despite documented poorer outcomes and higher mortality rates for the former, along with varying rates (1.6-71%) of incidence. Well-defined clinical guidelines and protocols for patient management related to type II MI are still scarce [5-9]. The main objective of our study was to outline the predisposing factors, precipitating elements, and possible outcomes of type II MI.

## Materials and methods

Study design

This prospective study was carried out at the Amrita Institute of Medical Sciences, Kochi, from January 1 to December 31, 2019 (12 months). The inclusion criterion was as follows: patients of ages 10-99 years with a final diagnosis of MI based on the levels of cardiac biomarkers and electrocardiography (ECG). Exclusion criteria were previous history of coronary angiography, thrombolysis, percutaneous coronary intervention (PCI), and any non-ischemic condition producing elevations in cardiac biomarkers.

Data collection

A total of 59 patients met the inclusion criteria during the study period and were included in the study. The demographic features (age and gender), cardiac biomarker levels [highly sensitive troponin I (Hs-Trop I) and creatine kinase myocardial band (CK-MB)], comorbidities [diabetes mellitus, hypertension, dyslipidemia, renal pathology, previous acute coronary syndrome (ACS), chronic obstructive pulmonary disease (COPD), peripheral vascular disease, chronic liver disease, and active malignancy], precipitating factors (sepsis, anemia, dyselectrolytemia, respiratory failure, arrhythmia, and postoperative stress), foci of sepsis (lower respiratory tract, hematogenous, urinary tract, and abdomen), and outcomes of type II MI (heart failure, arrhythmia, and death) were noted in a preformed proforma.

We only included patients with significant anemia (hemoglobin of <10 g/dL), capable of producing coronary insufficiency. Derangements in serum electrolytes were limited to ones directly related to arrhythmogenesis such as hypokalemia/hyperkalemia (K+ of <3 mEq/L or >5.5 mEq/L), hypocalcemia/hypercalcemia (Ca^2+ ^of <8.5 mEq/L or >12 mEq/L), and hypomagnesemia/hypermagnesemia (Mg of <1.5 mEq/L or >2.5 mEq/L).

Data analysis

Data were entered and analyzed on SPSS Statistics version 17 (IBM, Armonk, NY). The mean for age and cardiac biomarkers, and the frequency and percentages for gender, comorbidities, precipitating factors, foci of sepsis, and the outcomes of type II MI were calculated.

## Results

**Figure 1:**
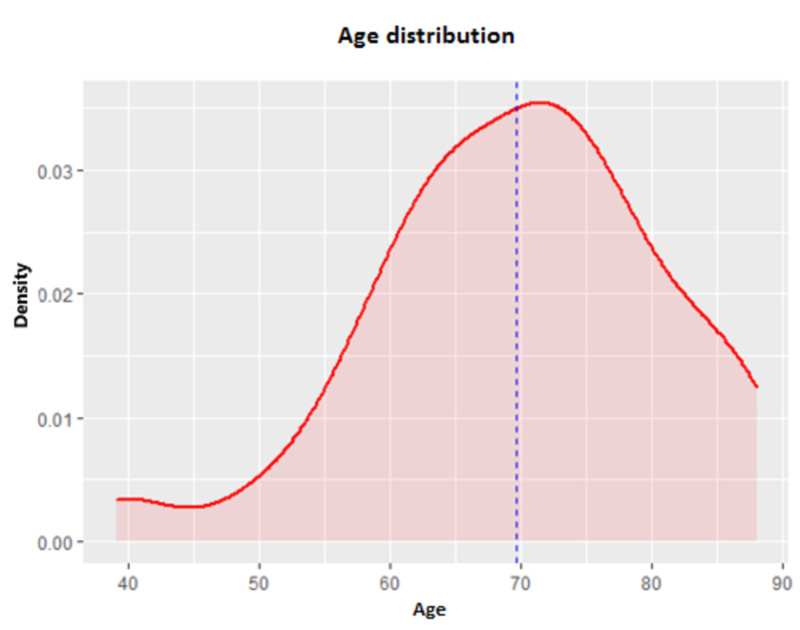
Age distribution of the patients

Patient age varied substantially, ranging from 39 to 88 years, with an average age of 69.66 years, as shown in Figure 1.

**Figure 2:**
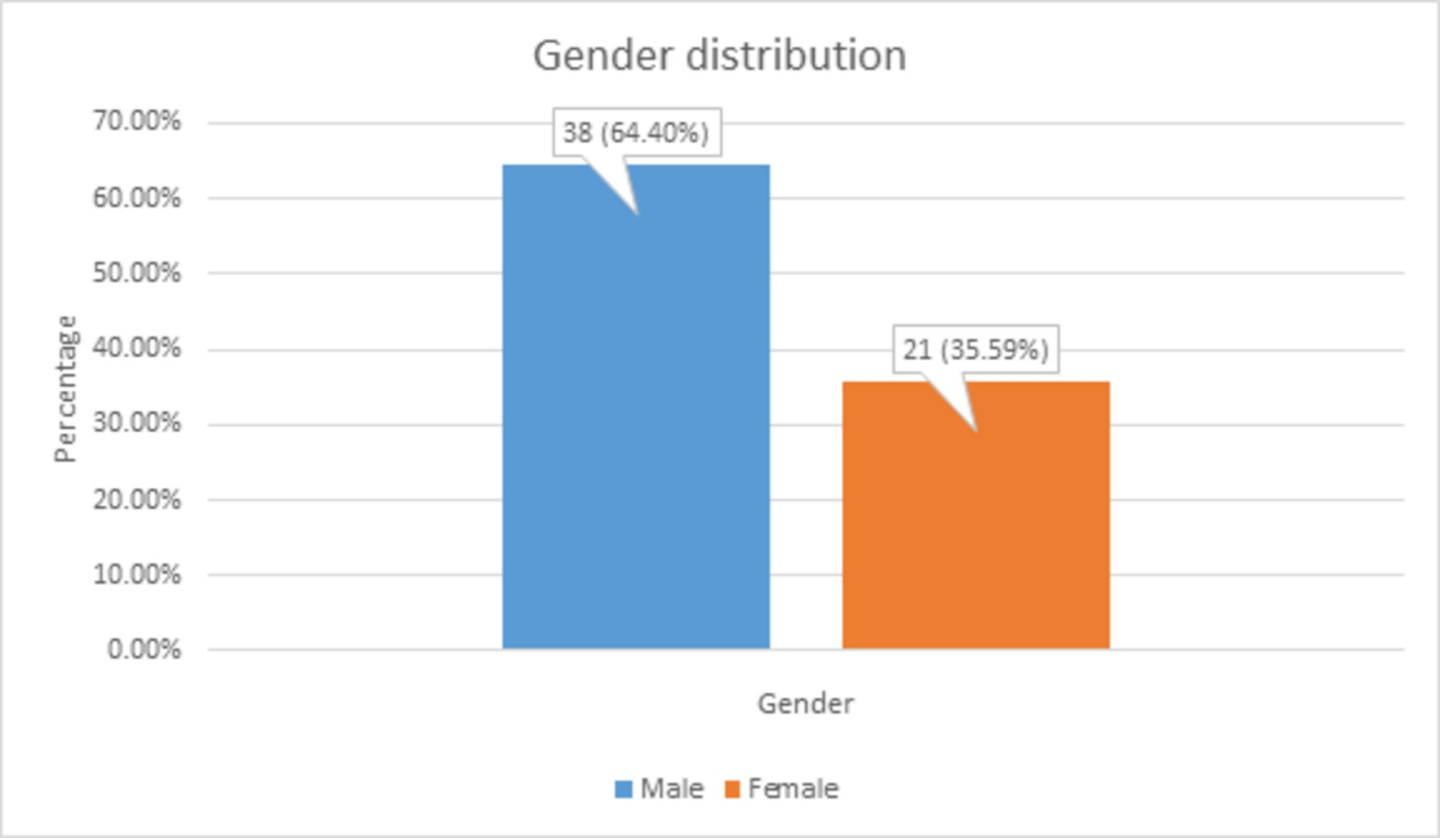
Gender distribution among the patients

There were 38 (64.4%) males and 21 (35.59%) females, contradictory to some studies that have reported a higher incidence in females, as shown in Figure 2.

**Figure 3:**
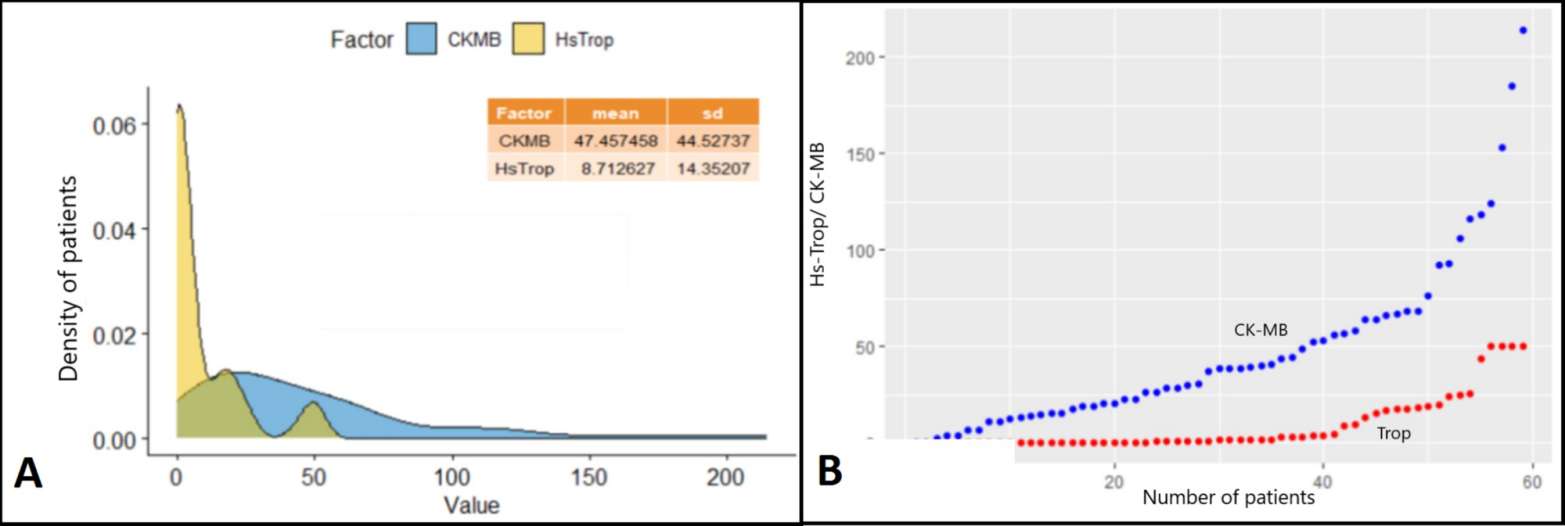
Distribution of cardiac biomarkers Panel A shows the mean elevation of CK-MB to a value of 47.457 IU/L and Hs-Trop I to a value of 8.712 ng/mL. Panel B shows the distribution of values of elevation, with the blue dots denoting CK-MB and red dots denoting Hs-Trop I. The cutoff for Hs-Trop I was set at 50 ng/mL CK-MB: creatine kinase myocardial band; Hs-Trop I: highly sensitive troponin I

Myocardial ischemia was characterized by the elevation of cardiac biomarkers, namely Hs-Trop I and CK-MB. Values were widely distributed and did not correlate with the severity of the disease. The correlative distribution of the values of cardiac biomarkers is shown in Figure 3.

All comorbidities were recorded and independently analyzed with a view to assessing which comorbidities were directly linked with an increased incidence of type II MI, as shown in Table 1.

**Table 1:** Distribution of comorbidities among the patients Diabetes mellitus and hypertension were the most common underlying patient comorbidities followed by dyslipidemia. This pattern is similar to the comorbidities associated with type I MI MI: myocardial infarction

Predisposing factor/comorbidity	Number of patients	Percentage
Diabetes mellitus	44	74.57%
Hypertension	41	69.49%
Dyslipidemia	38	64.4%
Renal pathology	21	35.59%
Previous acute coronary syndrome	20	33.38%
Chronic obstructive pulmonary disease	15	25.42%
Peripheral vascular disease	12	20.3%
Chronic liver disease	9	15.25%
Active malignancy	7	11.86%

**Figure 4:**
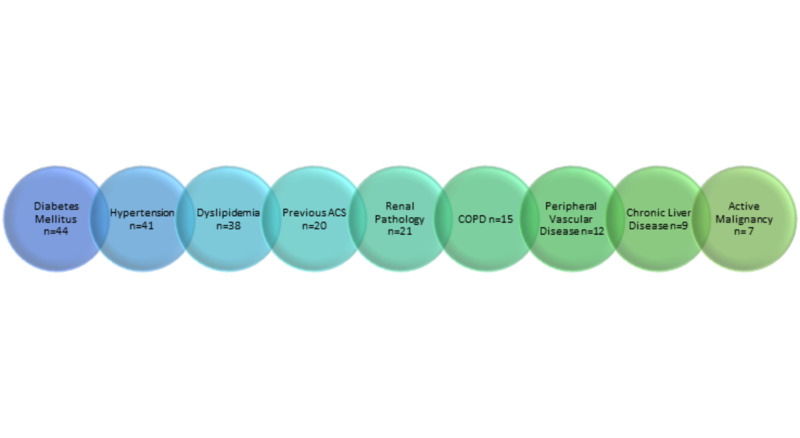
Overlap between comorbidities among the patients ACS: acute coronary syndrome; COPD: chronic obstructive pulmonary disease

There was a significant overlap between the comorbidities, with most patients having more than two diseases, as shown in Figure 4.

There were 33 (55.93%) patients with sepsis, 31 (52.4%) patients with significant anemia, 29 (49.1%) patients with electrolyte imbalance, 19 (32.2%) patients with respiratory failure, 16 (27.11%) patients with arrhythmia (tachyarrhythmia/bradyarrhythmia), and two (3.3%) patients with postoperative (non-cardiothoracic) stress. The distribution of precipitating factors of coronary insufficiency is shown in Table 2.

**Table 2:** Factors precipitating coronary insufficiency

Factors	Number of patients	Percentage
Sepsis	33	55.93%
Anemia	31	52.4%
Dyselectrolytemia	29	49.1%
Respiratory failure	19	32.2%
Arrhythmia	16	27.11%
Postoperative stress	2	3.3%

The focus of sepsis was the lower respiratory tract in 14 (42.42%) patients, hematogenous in 11 (33.33%) patients, genitourinary in eight (24.24%) patients, and abdominal in six (18.18%) patients, as shown in Table 3.

**Table 3:** Different foci of sepsis among the patients

Focus of sepsis	Number of patients	Percentage
Lower respiratory tract	14	42.42%
Hematogenous	11	33.33%
Urinary tract	8	24.24%
Abdomen	6	18.18%

**Figure 5:**
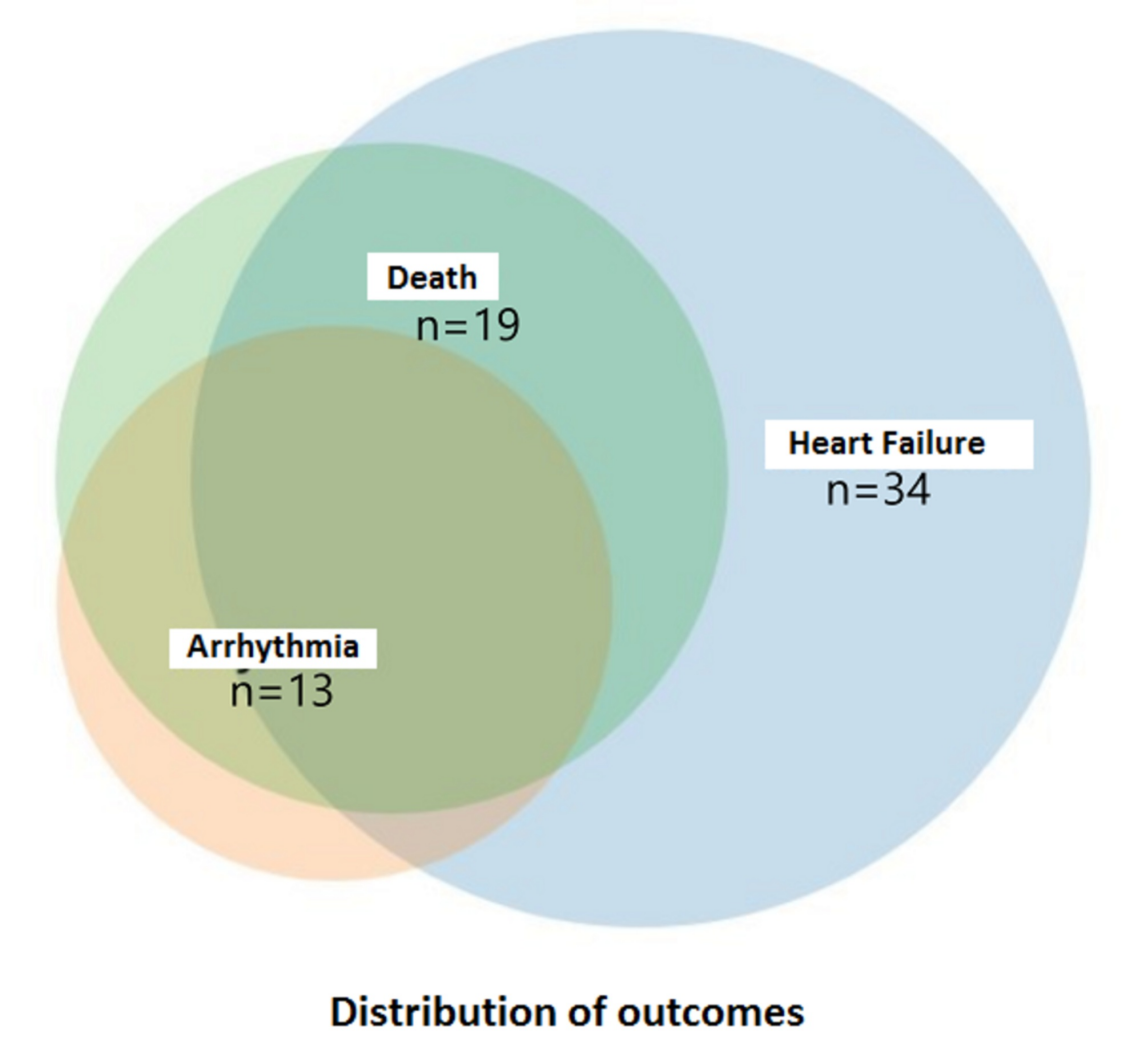
Distribution of outcomes of type II myocardial infarction

A majority of patients experienced heart failure, as evidenced by clinical features and elevation of N-terminal-pro B-type natriuretic peptide (NT-pro BNP), followed by fatal arrhythmias and death. The distribution of outcomes of type II MI is shown in Figure 5 and Table 4.

**Table 4:** Outcomes following type II myocardial infarction

Outcome	Number of patients	Percentage
Heart failure	34	57.62%
Arrhythmia	13	22.03%
Death	19	32.20%
Death following heart failure	8	42.21%
Death following arrhythmia	2	10.5%
Death following both	9	47.36%

## Discussion

This study showed that there is a high risk of type II MI in anemic patients, patients with electrolyte abnormalities, patients with peripheral vascular disease, and those with features of sepsis. It also revealed that patients going into ventricular failure often have multiple precipitating factors, each one probably compounding the effect of the other on myocardial vascular insufficiency. The primary goal of defining these parameters is to differentiate this clinical entity from its better-known counterpart, the type I MI. Numerous studies have outlined and shown increased morbidity and mortality for type II MI, with the precipitating factors themselves carrying significant mortality rates.

MI has always occupied a venerable place among physicians over the years. Ever since its description in the 18th century by John Hunter, clinical features and diagnosis along with treatment protocols for MI have continued to divide opinion even at the highest tiers of clinical and interventional medicine. This is due to the fact that despite rapidly advancing techniques of detection and diagnosis, a large number of cases are inadequately treated in the face of an increasing number of cases in India [4].

The definition of MI has been associated with much debate and speculation. According to the latest fourth universal definition of MI, the clinical entity is broadly divided into five subtypes [5].

Type I MI

Type I is the most common type of MI. Ischemia occurs due to an acute change in pre-existing atherosclerotic plaque, which can occur in the form of plaque erosion, thrombosis, or hemorrhage.

Type II MI

**Figure 6:**
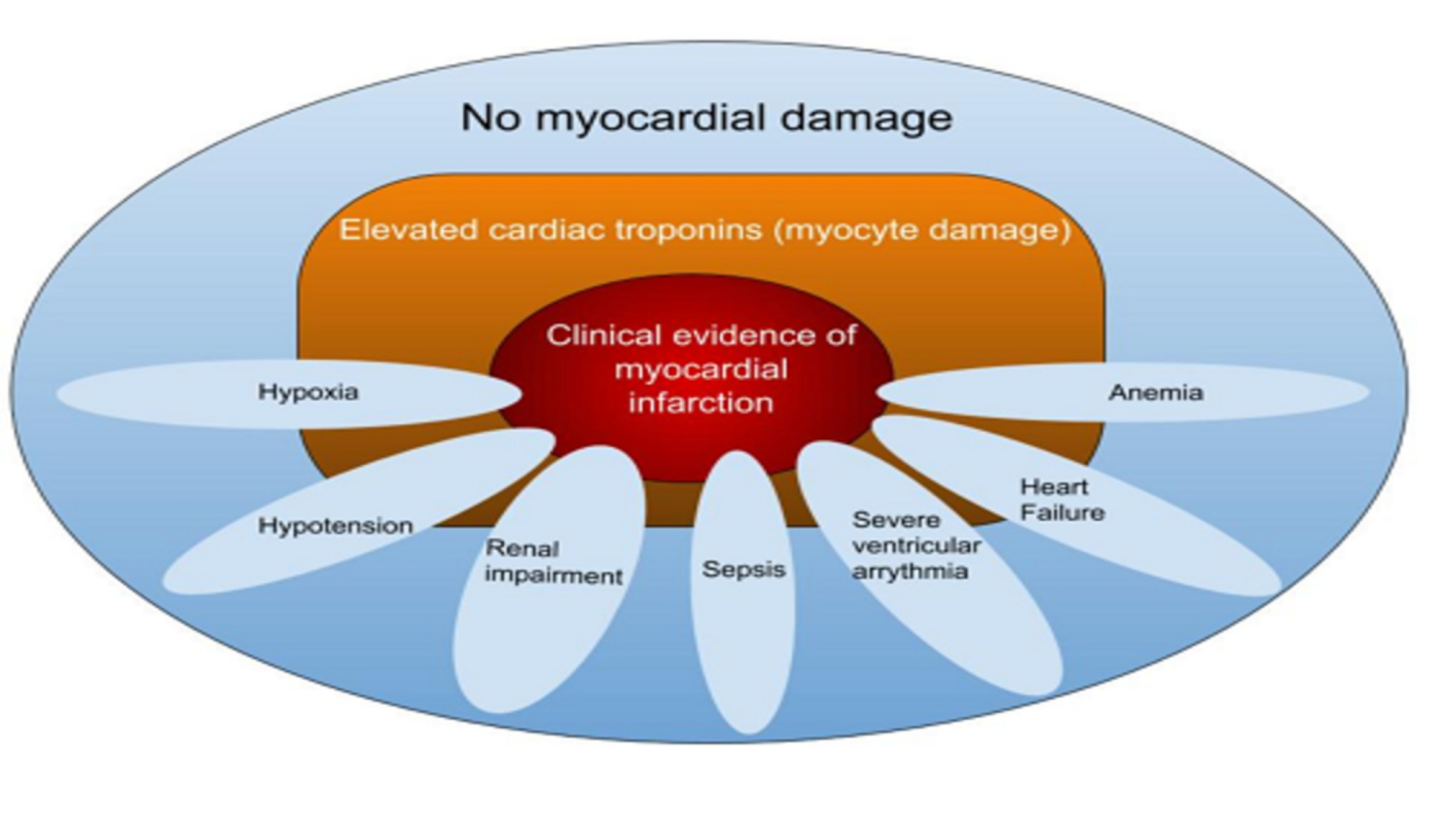
Factors precipitating coronary insufficiency

Myocardial ischemia generally occurs due to a mismatch in oxygen supply in relation to myocardial metabolic demand. It can occur in the presence of stable atherosclerotic plaque (as per the 2012 revision in the definition) as long as there is no acute plaque change. Plaque rupture changes the classification from type II to I. Infrequently, a type I may superimpose on an initial type II MI. Type II MI is associated with numerous risk factors, the only inclusive criteria being a direct causality to an increase in myocardial oxygen demand. Some of the more common causes are sepsis, arrhythmia (sustained tachyarrhythmia or severe bradyarrhythmia), anemia, severe left ventricular failure, or sudden hypotension. Some other rare causes include coronary embolism (non-plaque-related), coronary dissection, or coronary spasm (Figure 6).

Type II MI is defined as the detection of a rise and/or fall of cardiac troponin values with at least one value above the 99th percentile of the upper reference limit, and evidence of an imbalance between myocardial oxygen supply and demand unrelated to coronary thrombosis, requiring at least one of the following features: symptoms of acute myocardial ischemia, new ischemic ECG changes, development of pathological Q waves, or imaging evidence of new loss of viable myocardium or new regional wall motion abnormality in a pattern consistent with ischemia [5].

Type III MI

This diagnosis is made when death occurs as a result of strong clinical suspicion or events suggestive of acute MI; it is a retrospective diagnosis. Diagnosis is often made when the cause of death is unknown and has to be evaluated. Elevation in serum cardiac biomarker levels is not a prerequisite, though it is commonly seen. Strong electrophysiological change suggestive of acute myocardial ischemia or events leading to cardiac arrest (ventricular fibrillation, severe hyperkalemic pattern, features of digoxin toxicity, etc.) are mandatory for diagnosis.

Type IV MI

This type of MI is associated with coronary revascularization procedures. Based on the latest criteria, the reported incidence of this condition is between 14.5-32% [10]. A baseline, stable troponin value must be established showing a rising trend above the 99th percentile to make a diagnosis of type IV acute MI. Small elevations in cardiac troponins are common post-procedurally, possibly due to surgical myocardial manipulation. Type IV MI is further subdivided into three subtypes.

Type IVa

This is defined as PCI-associated MI. A rise in troponin of greater than 20% from the baseline, with this new value being greater than five times the upper limit of the reference value, is mandatory for diagnosing post-PCI MI. There must also be corroboratory evidence of recent myocardial ischemia in the form of ECG changes suggestive of ischemia, or imaging findings. Infarction may also occur as a procedure-related complication that is known to produce myocardial ischemia, such as coronary dissection, distal embolization, and thrombosis of major/side epicardial arteries.

Type IVb

This term is used to describe MI associated with stent/scaffold thrombosis. The criteria are the same as for type I except that this occurs in relation to PCI. It is further subclassified into acute (<24 hours), subacute (1-30 days), late (31 days-one year), and very late (>one year) based on the onset after stent placement.

Type IVc

This type is defined as ischemia due to restenosis of the coronary vessel following PCI. The restenosis of the stent may be due to secondary atherosclerotic processes or intimal neovascularization. This is a diagnosis of exclusion; it is made only when no other culprit lesion can be picked up during angiography. The rise of troponin must be >99th percentile of the upper reference limit from the baseline.

Type V MI

This type is defined as MI associated with coronary artery bypass grafting (CABG). Usually, a mild degree of myocardial ischemia following CABG is common, which is attributed to ischemia secondary to cardiac bypass. This degree of troponin rise is higher in on-the-pump CABG compared to off-the-pump surgeries. The damage is primarily due to reperfusion injuries that occur while weaning the patient off the cardiopulmonary bypass. Since a minor myocardial injury is expected, the cutoff for troponin rise is raised, and the detected troponin level must be >10 times the upper reference limit. It must reach this value from a normal baseline (<99th percentile) within 48 hours. In addition, the values must also show a rising trend to establish this diagnosis. The troponin rise must also be accompanied by corroboratory evidence of injury as evidenced by ECG changes or cardiac imaging.

In our study, we found that sepsis (55.93%) was the most common factor precipitating coronary insufficiency, the same condition itself having a pre-mortality rate ranging from 17-41%, with sepsis-related deaths showing an increasing trend annually [11-13].

## Conclusions

Type II MI is generally associated with a poor prognosis. Patients suffering from type II MI can have multiple comorbidities, with diabetes mellitus and hypertension being the most frequent among them. In our study, sepsis was found to be the most common risk factor precipitating coronary insufficiency, the focus of which predominantly being the lower respiratory tract. Anemia and dyselectrolytemia were the concomitant factors furthering the degree of coronary insufficiency. Such patients had a high incidence of ICU stay and needed prolonged intensive care and multiple therapeutic interventions. A high index of suspicion and clinical awareness are the keys to early diagnosis and intervention for type II MI. The management of type II MI is primarily centered around treating the factors precipitating coronary insufficiency.

## References

[1] Ward MJ, Kripalani S, Zhu Y, Storrow AB, Dittus RS, Harrell FE Jr, Self WH (2015). Incidence of emergency department visits for ST-elevation myocardial infarction in a recent six-year period in the United States. Am J Cardiol.

[2] Gupta R, Mohan I, Narula J (2016). Trends in coronary heart disease epidemiology in India. Ann Glob Health.

[3] Task Force on the management of ST-segment elevation acute myocardial infarction of the European Society of Cardiology (ESC), Steg PG, James SK (2012). ESC guidelines for the management of acute myocardial infarction in patients presenting with ST-segment elevation. Eur Heart J.

[4] McCarthy BD, Beshansky JR, D’Agostino RB, Selker HP (1993). Missed diagnoses of acute myocardial infarction in the emergency department: results from a multicenter study. Ann Emerg Med.

[5] Thygesen K, Alpert JS, Jaffe AS (2019). Fourth universal definition of myocardial infarction (2018). Eur Heart J.

[6] El-Haddad H, Robinson E, Swett K, Wells GL (2012). Prognostic implications of type 2 myocardial infarctions. World J Cardiovasc Dis.

[7] Melberg T, Burman R, Dickstein K (2010). The impact of the 2007 ESC-ACC-AHA-WHF universal definition on the incidence and classification of acute myocardial infarction: a retrospective cohort study. Int J Cardiol.

[8] Saaby L, Poulsen TS, Hosbond S (2013). Classification of myocardial infarction: frequency and features of type 2 myocardial infarction. Am J Med.

[9] Chapman AR, Adamson PD, Mills NL (2017). Assessment and classification of patients with myocardial injury and infarction in clinical practice. Heart.

[10] Skeik N, Patel DC (2007). A review of troponins in ischemic heart disease and other conditions. Int J Angiol.

[11] Fleischmann C, Thomas-Rueddel DO, Hartmann M (2016). Hospital incidence and mortality rates of sepsis: an analysis of hospital episode (DRG) statistics in Germany from 2007 to 2013. Dtsch Arztebl Int.

[12] Fleischmann C, Scherag A, Adhikari NK (2016). Assessment of global incidence and mortality of hospital-treated sepsis. current estimates and limitations. Am J Respir Crit Care Med.

[13] Dombrovskiy VY, Martin AA, Sunderram J, Paz HL (2007). Rapid increase in hospitalization and mortality rates for severe sepsis in the United States: a trend analysis from 1993 to 2003. Crit Care Med.

